# Equol Pretreatment Protection of SH-SY5Y Cells against Aβ (25–35)-Induced Cytotoxicity and Cell-Cycle Reentry via Sustaining Estrogen Receptor Alpha Expression

**DOI:** 10.3390/nu11102356

**Published:** 2019-10-03

**Authors:** Meng-Chao Tsai, Shyh-Hsiang Lin, Kiswatul Hidayah, Ching-I Lin

**Affiliations:** 1 Department of Psychiatry, Taoyuan General Hospital, Taoyuan 33004, Taiwan; mctsai1981@gmail.com; 2 School of Nutrition and Health Sciences, Taipei Medical University, Taipei 11042, Taiwan; lin5611@tmu.edu.tw (S.-H.L.); kiswatul.hidayah@gmail.com (K.H.); 3 Master Program in Food Safety, Taipei Medical University, Taipei 11042, Taiwan; 4 Research Center of Geriatric Nutrition, Taipei Medical University, Taipei 11042, Taiwan; 5 Department of Nutrition and Health Sciences, Kainan University, Taoyuan 33857, Taiwan

**Keywords:** S-equol, 17β-estradiol, estrogen receptor alpha, cell cycle, β-Amyloid, Alzheimer’s disease

## Abstract

β-amyloid formation in the brain is one of the characteristics of Alzheimer’s disease. Exposure to this peptide may result in reentry into the cell cycle leading to cell death. The phytoestrogen equol has similar biological effects as estrogen without the side effects. This study investigated the possible mechanism of the neuron cell-protecting effect of equol during treatment with Aβ. SH-SY5Y neuroblastoma cells were treated with either 1 μM S-equol or 10 nM 17β-estradiol for 24 h prior to 1 μM Aβ (25–35) exposure. After 24 h exposure to Aβ (25–35), a significant reduction in cell survival and a reentry into the cell cycle process accompanied by increased levels of cyclin D1 were observed. The expressions of estrogen receptor alpha (ERα) and its coactivator, steroid receptor coactivator-1 (SRC-1), were also significantly downregulated by Aβ (25–35) in parallel with activated extracellular signal-regulated kinase (ERK)1/2. However, pretreatment of cells with S-equol or 17β-estradiol reversed these effects. Treatment with the ER antagonist, ICI-182,780 (1 μM), completely blocked the effects of S-equol and 17β-estradiol on cell viability, ERα, and ERK1/2 after Aβ (25–35) exposure. These data suggest that S-equol possesses a neuroprotective potential as it effectively antagonizes Aβ (25–35)-induced cell cytotoxicity and prevents cell cycle reentry in SH-SY5Y cells. The mechanism underlying S-equol neuroprotection might involve ERα-mediated pathways.

## 1. Introduction

Neuronal cell death is an important feature of human neurodegenerative diseases such as Alzheimer’s disease (AD). This cell death is considered to occur as a consequence of aberrant activation of the cell cycle in neurodegeneration [1]. Under normal conduction, the cell cycle is tightly controlled by specific regulatory proteins. For instance, cyclins and cyclin-dependent kinases (CDKs) are two key classes of regulatory molecules that determine a cell’s progress in the cell cycle [2]. As a key regulator of the G1-S transition, cyclin D1 interacts with CDK4 to form the cyclin D1-CDK4 complex and moves to the nuclei, thereby promoting cell cycle progression. Normal adult neuron cells never reenter the cell cycle (but stay in the G_0_ stage) and are thus recognized as permanently postmitotic cells [3]. Conversely, neurons reenter the cycle, undergo DNA replication, and die after they are exposed to DNA-damaging agents, oxidative stress, or certain neurotoxins such as beta-amyloid (Aβ) aggregates [3]. The Aβ peptide is the major component of senile plaque derived from the Aβ precursor protein (APP); this peptide is a neuropathological hallmark of AD [4]. There are numerous different Aβ species including Aβ (1–40), Aβ (1–42), and Aβ (25–35). The Aβ (25–35) fragment is universally used in research as it has been found to elicit profound toxic manifestations in elderly people and to physiologically play a role in AD [5]. It has been previously shown that cell cycle activation accompanied by the upregulation of cyclin D1 in primary cultured rat cortical neurons was observed in response to exposure to Aβ (25–35) and that such activation was followed by apoptotic neuronal death [6]. To elucidate the possible intracellular signaling pathway involved in the activation of the cell cycle by Aβ, extracellular signal-regulated kinase (ERK) 1/2-related pathways are the major focus of the present study because there is evidence of the involvement of ERK1/2 activation in Aβ-induced neuronal cell death [7]. It has been documented that activation of ERK 1/2 appears to be critical for G1 to S phase progression in cell cycle regulation [8]. A previous study showed that the overexpression of ERK 1/2 in cells exposed to Aβ was followed by an elevation in cyclin D1 expression, which resulted in changes in the cell-cycle distribution, particularly in the G1-S phase [9]. 

ERK1/2 is also the target of the regulatory action of estrogen and its regulation requires interaction with the known estrogen receptors (ERs), ERα and ERβ [10]. In addition to the reproductive system, both ERα and ERβ are broadly expressed in nonreproductive systems including the central nervous system [11]. Particularly, brain regions such as the hypothalamus, amygdala, and hippocampus appear to have distinct expression patterns of both ER subtypes [12]. Although it is recognized that ERβ is the predominant receptor in the hippocampus, where its absence has an impact on memory and cognitive function [13], ERα co-exists, and its coregulation may be important for ERβ to fulfill its cellular roles [14]. In other words, ERβ collaborating with ERα in its molecular actions is crucial for estrogen-mediated beneficial effects on hippocampus-dependent memory and cognition. The ERα subtype is of particular interest in the present study as it exhibits stronger transcription activity than ERβ and thus appears to be functionally superior to ERβ in the modulation of age-related memory decline [13,14,15]. It is noteworthy that ERα diminishing in the hippocampus with age leads to a decrease in the relative expression of ERα and ERβ, and nuclear ERα-mediated effects, all of which are putative molecular mechanisms for age-related memory decline in the presence of low estrogen levels [13]. In this regard, the molecular actions of both ER subtypes have been reported to be involved in the neuroprotection of estrogen against the pathogenic processes of AD [16]. Evidence suggests that estrogen is capable of protecting against Aβ-induced toxicity through ERα-mediated signaling pathways [17]. Moreover, the other major neuropathological hallmark of AD is intracellular aggregates of hyperphosphorylated Tau protein, which has recently been found to interact with ERα potentiating the reduction of ERα’s transcriptional activity [18]. SRC-1 is one of the nuclear receptor coactivators which enhance the transcriptional activity of ERs to manipulate the relevant molecular events [19]. Studies performed in a human astrocytoma cell line demonstrated that estradiol treatment increased the cell number through the mediation of ERα, whereas the coactivator silencing by RNA interference of SRC-1 was able to block this effect [19].

Equol is a metabolite of daidzein, one of the major isoflavones in soybean food products, and is known as an ERs agonist [20]. Equol is capable of inducing transcriptional responses, especially through the binding of ERα [21]. The oral bioavailability of equol in humans seems to be high, resulting in a plasma concentration of 0.4~2 μM after taking a single bolus of 2 mg of equol [22]. Consumption of phytoestrogens has been found to avoid many side effects from estrogens [23]. Intriguingly, equol has been shown to be a promising neuroprotectant in in vitro models, and its neuroprotective effects are exerted through anti-neuroinflammatory mechanisms with the regulation of relevant signaling pathways at molecular levels [24]. However, whether the cell cycle regulatory event and ER-dependent signaling pathways involve the neuroprotective properties of equol remains an enigma. Thus, in this study, we investigated the effects of equol on protecting SH-SY5Y cells against Aβ-induced perturbations and the cellular mechanisms underlying equol’s neuroprotective action in cell cycle events and ER pathways.

## 2. Materials and Methods 

### 2.1. Cell Culture

Human SH-SY5Y neuroblastoma cells were cultured at 37 °C and 5% CO_2_ in Dulbecco’s modified Eagle medium (DMEM) (Invitrogen™, Life Technologies, Grand Island, NY, USA) mixed with F12 (Invitrogen™, Life Technologies, Grand Island, NY, USA), 10% fetal bovine serum (FBS) (Biowest LLC, Miami, FL, USA), and glutamine (Biological Industries, Kibbutz Beit Haemek, Israel). The medium was changed twice per week. Cells were grown to 80% confluence before treatment.

### 2.2. Treatments

Aβ (25–35) (Sigma Aldrich, St. Louis, MO, USA) was dissolved in sterile distilled water at a concentration of 1 mM, then incubated in a capped vial at 37 °C for 5 days to allow formation of the aggregated form. It was then stored frozen at −20 °C until use. 17β-Estradiol, S-equol, and ICI-182,780 (all from Cayman Chemical, Ann Arbor, MI, USA) were dissolved in 99.5% ethanol to make stock solutions, which were used for experiments at a final concentration of 10 nM for estradiol and 1 μM for equol and ICI-182,780 in culture medium. It should be noted that no cytotoxic effect of the vehicle (99.5% ethanol) *per se* on cells was observed via the analysis of cell viability in our preliminary experiments that were conducted to determine the appropriate concentrations of the aforementioned treatments for the present study.

To induce cell death, cells were incubated with (Aβ) or without (C) 1 μM Aβ (25–35) for 24 h. To study the effects of estradiol (E2) and equol (Eq), cells were preincubated with estradiol (E2 + Aβ) or equol (Eq + Aβ) for 24 h prior to Aβ (25–35) exposure. Estradiol was used as a positive control and ICI-182,780 was used as an ER antagonist. It was added 1 h before the estradiol or equol treatment.

### 2.3. Cell Viability Analysis

Cell viability was assessed using a modified 3-[4,5-dimethylthiazol-2]-2,5 diphenyltetrazolium bromide (MTT) assay (Sigma, St. Louis, MO, USA). Cells were seeded in 24-well dishes at a seeding density of 2 × 10^5^ cells/well. After treatment, 300 μL of the MTT solution (5 mg/mL) was added to each well and incubated at 37 °C for 3 h. After removing the culture medium, 250 μL of dimethyl sulfoxide (DMSO) was added to each well to dissolve the formazan, and then 200 μL of the solution was moved to a 96-well dish. The optical density was measured at 570 nm using a microplate reader. The absorbance of the control group was considered to have 100% cell viability.

### 2.4. Protein Extraction and Quantification

After treatment, cells were harvested, washed three times with PBS, and lysed using a cold RIPA lysis buffer supplemented with a protease inhibitor and an EDTA solution (Thermo, Hudson, NH, USA) at a ratio of 100:1:1, then centrifuged at 13,000 rpm and 4 °C for 30 min. The supernatant was collected, and the protein concentration was estimated with a BCA Protein Assay Kit (Sigma, St. Louis, MO, USA) using BSA as the standard.

### 2.5. Cell-Cycle Analysis

Cells (8 × 10^5^) were seeded in 6-well dishes. After treatment, cells were trypsinized, washed in PBS, and centrifuged at 2000× *g* at 25 °C for 5 min, and then they were washed with PBS at least twice. Cells were fixed in 70% ethanol overnight. Before removing the ethanol, samples were centrifuged at 11 °C and 2200× *g* for 10 min. The pellet was then resuspended in 200 μL of DNA extraction buffer (containing 192 mL 0.2 M Na_2_HPO_4_ and 8 mL 0.1 M citric acid at pH 7.8) and incubated for 30 min at 37 °C. PI dye (200 μL, containing 0.1% Triton-X100, 100 μg/mL RNase-A, and 80 μg/mL PI in PBS) was added, gently mixed, and incubated for 30 min at room temperature in the dark. After removing the PI dye, samples were resuspended with 1 mL of cold PBS prior to analysis by flow cytometry.

### 2.6. Western Blot Analysis

A western blot analysis was performed to examine the expression levels of the proteins. Equal quantities (30 μg) of protein were separated by 10% sodium dodecyl sulfate polyacrylamide gel electrophoresis (SDS-PAGE) and then transferred onto nitrocellulose membranes. After transfer, membranes were blocked with Tris-buffered saline (TBS) containing 0.1% Tween-20 (TBST) and 5% non-fat-milk for 1 h. The membranes were then incubated with specific primary antibodies (Cell Signaling Technology, Danvers, MA, USA): Anti-cyclin D1 (1:1000), anti-p-ERK 1/2 (1:1000), anti-ERK 1/2 (1:1000), anti-ERα (1:1000), anti-SRC-1 (1:1000), and anti-β-actin (1:5000) overnight at 4 °C. After washing three times with TBST for 30 min, membranes were incubated with an anti-rabbit (1:80000) or anti-mouse (1:5000) immunoglobulin G (IgG) secondary antibody (Sigma) for 1 h, and then washed with TBST three times for 30 min. Immunoreactive proteins were detected by enhanced chemiluminescence (ECL) (Bionovas, Toronto, Canada) Western blot detection system.

### 2.7. Statistical Analysis

Data are shown as the mean and standard deviation (SD). Statistical comparisons were performed using SAS 9.3 (Cary, NC, USA). One-way analysis of variance (ANOVA) and least squared difference (LSD) post-hoc analysis of multiple comparisons were used. The statistical significance was accepted at *p* < 0.05.

## 3. Results

### 3.1. Cell Viability

**Figure 1:**
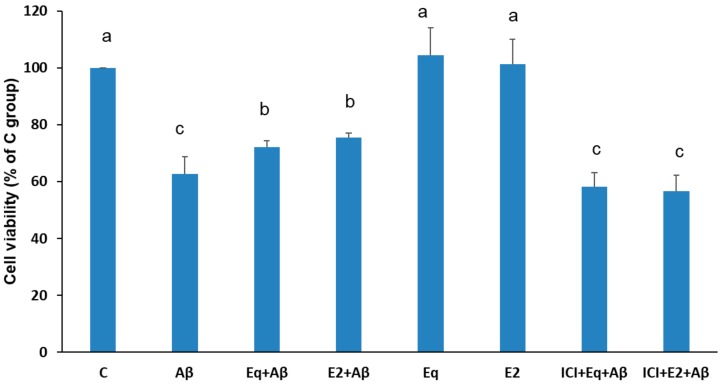
Cell viability of SH-SY5Y cells assessed with a modified 3-[4,5-dimethylthiazol-2]-2,5 diphenyltetrazolium bromide (MTT) assay. Data were analyzed by one-way ANOVA followed by the LSD post-hoc test and are representative of three independent experiments (*n* = 3). Values are presented as the mean + SD. Bars with different letters significantly differ at a level of *p* < 0.05. Eq, S-equol; E2, 17β-estradiol; ICI, ICI-182,780.

As shown in Figure 1, the cell viability of the Aβ group decreased to 62.6% compared to the C group (*p* < 0.05), suggesting that Aβ (25–35) is cytotoxic to SH-SY5Y cells in the present study. After pretreatment with equol (Eq + Aβ), the cell viability was significantly increased by 9.6% compared to the Aβ group, and the same effect was observed in the E2 group which exhibited increased cell viability of up to 12.9% compared to the Aβ group (*p* < 0.05). No cytotoxic effect on cells was found from the treatments of 17β-estradiol (E2) and S-equol (Eq). These findings indicate that Eq, like E2, had the potential to provide the neuroprotective effects against Aβ cytotoxicity *in vitro*. Moreover, in order to confirm that the neuroprotective effects of S-equol and 17β-estradiol against Aβ (25–35) cytotoxicity are mediated by the estrogen receptors, cells were pretreated with 1 μM ER antagonist ICI-182,780 for 1 h prior to Eq or E2 treatment. In the presence of Aβ (25–35), pretreatment with ER antagonism of ICI-182,780 prior to Eq or E2 treatments significantly abolished their effects on SH-SY5Y cell viability (*p* < 0.05). These results suggest that Eq and E2 antagonized the reduced cell viability-induced by Aβ (25–35), at least in part, by mediating the ERs.

### 3.2. Estrogen Receptor Alpha (ERα) Protein Expression

**Figure 2:**
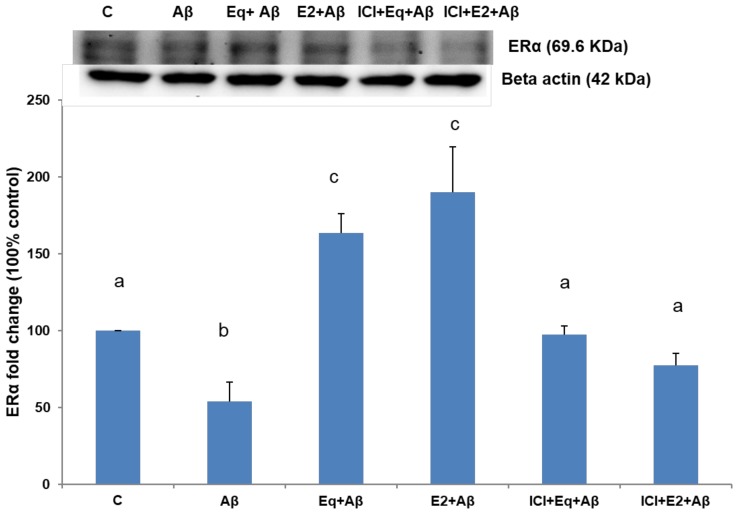
The estrogen receptor alpha (ERα) expressions of SH-SH5Y cells from different treatments. Data were analyzed by a one-way ANOVA followed by the LSD post-hoc test and are representative of three independent experiments (*n* = 3). Values are presented as the mean + SD. Bars with different letters are significantly different at *p* < 0.05.

Figure 2 shows that Aβ (25–35) alone markedly reduced the protein expression of ERα (*p* < 0.05), whereas pretreatments of Eq and E2 significantly attenuated the decreased protein expressions of ERα induced by Aβ (25–35) (*p* < 0.05). However, in the presence of ICI-182,780, the effects of the Eq and E2 pretreatments on ERα expression were significantly blocked (*p* < 0.05).

### 3.3. SRC-1 Protein Expression

**Figure 3:**
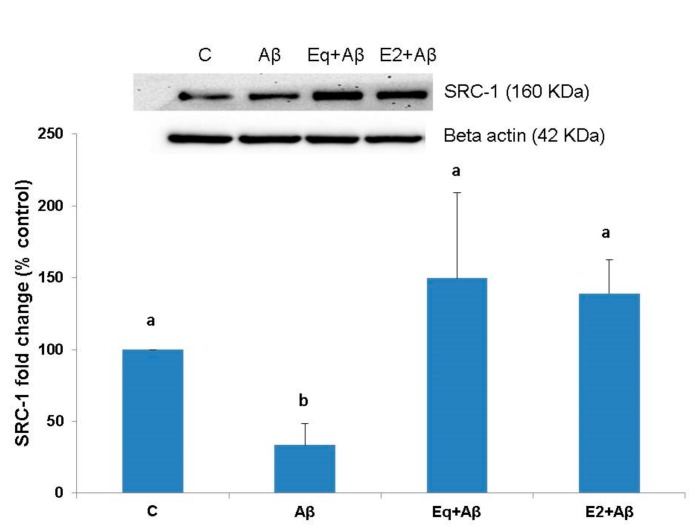
The SRC-1 expressions of SH-SH5Y cells from different treatments. Protein expressions were assessed by Western blotting. Data were analyzed by a one-way ANOVA followed by the LSD post-hoc test and are representative of three independent experiments (*n* = 3). Values are presented as the mean + SD. Bars with different letters are significantly different at *p* < 0.05.

Figure 3 shows the effects of the Eq and E2 pretreatments on the expression of the estrogen receptor coactivator SRC-1. Treatment of 1 μM Aβ (25–35) (Aβ) for 24 h significantly decreased the SRC-1 protein expression (*p* < 0.05). Pretreatment with either Eq or E2 significantly prevented Aβ (25–35)-induced reduction in SRC-1 protein expression (*p* < 0.05).

### 3.4. Cell Cycle

**Figure 4:**
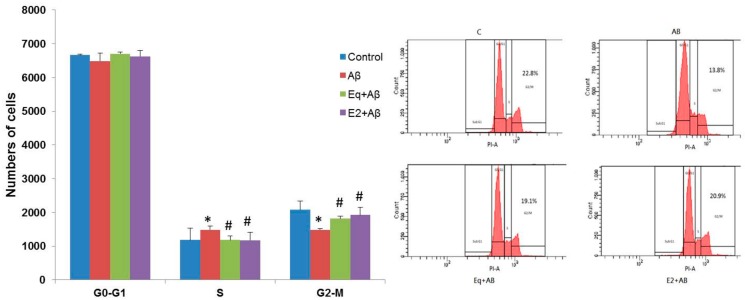
Cell cycle events of SH-SH5Y cells from different treatments. The cell cycle was assessed by PI staining with flow cytometry. Data were analyzed by one-way ANOVA followed by the LSD post-hoc test and are representative of three independent experiments (*n* = 3). Values are presented as the mean + SD. * *p* < 0.05 vs. the control; ^#^ *p* < 0.05 vs. the Aβ group.

Figure 4 shows the distribution of different phases of cell cycle. A significant increase of cells in the S phase in the Aβ group is observed compared to the C group as well as a concomitant reduction of cells in the G_2_/M phases of the cycle (Figure 4). This result indicates that SH-SY5Y cells exposed to Aβ (25–35) escaped from the G_2_/M phase. Pretreatment with either Eq (Eq + Aβ) or E2 (E2 + Aβ) showed a decreasing number of cells in the S phase and a significantly increasing number in the G_2_/M phase compared to that in the Aβ group (*p* < 0.05). Cell cycle analysis showed that the cell cycle profiles were markedly altered by the treatment of Aβ (25–35), and pretreatment with Eq or E2 significantly blocked Aβ (25–35)-induced changes in the cell cycle profiles of the SH-SY5Y cells.

### 3.5. Cyclin D1 Protein Expression

**Figure 5:**
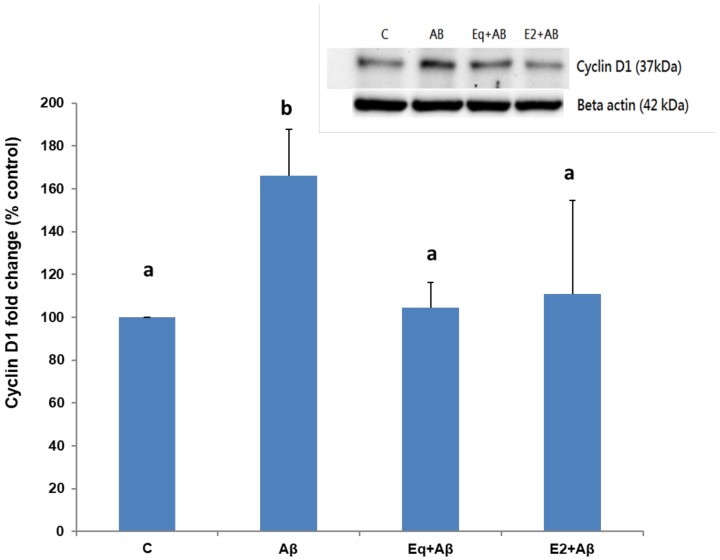
The cyclin D1 expressions of the SH-SH5Y cells from different treatments. Protein expressions were assessed by Western blotting. Data were analyzed by a one-way ANOVA followed by the LSD post-hoc test and are representative of three independent experiments (*n* = 3). Values are presented as the mean + SD. Bars with different letters are significantly different at *p* < 0.05.

Figure 5 shows the relative expressions of cyclin D1, a protein marker for the G_0_/G_1_ phase, in different treatments. The relative expression increased markedly after cells were treated with Aβ (25–35) (Aβ group) in comparison to the untreated cells (C group). A decreased level of expression was observed in cells which underwent 24-h Eq or E2 pretreatment (Eq + Aβ group and E2 + Aβ group, respectively), compared to the Aβ group (*p* < 0.05).

### 3.6. Activation of ERK 1/2

**Figure 6:**
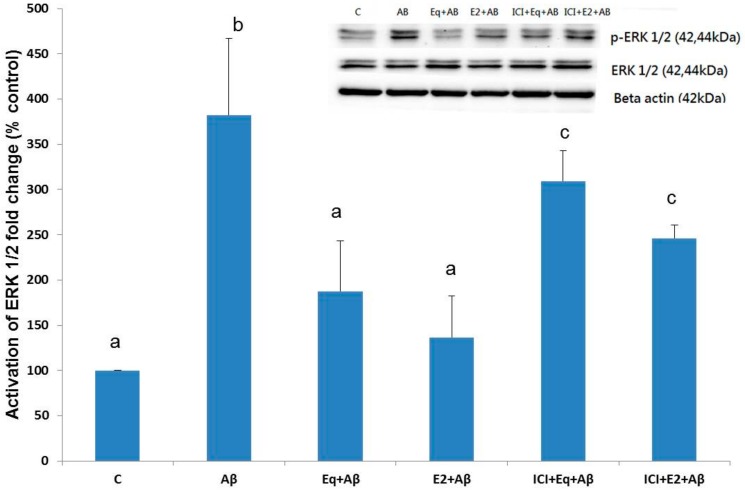
The phosphorylated (activated) ERK 1/2 expressions of SH-SH5Y cells from different treatments. Data were analyzed by a one-way ANOVA followed by the LSD post-hoc test and are representative of three independent experiments (*n* = 3). Values are presented as the mean + SD. Bars with different letters are significantly different at *p* < 0.05.

Figure 6 shows that Aβ (25–35) treatment significantly increased the expression of phosphorylated (p)-ERK 1/2 (*p* < 0.05). The pretreatments of Eq and E2 significantly prevented the Aβ (25–35)-induced activation of ERK 1/2 (*p* < 0.05). On the other hand, when the ER activity was inhibited by ICI-182,780, the effect of Eq and E2 on deactivation of ERK 1/2 was significantly reduced (*p* < 0.05).

## 4. Discussion

Evidence from previous clinical and experimental studies showed that estrogen replacement therapy may have beneficial effects on AD in postmenopausal women [25,26]. However, the use of estrogen as treatment is known to have side effects, such as the development of breast and endometrial cancers in women [23]. Phytoestrogens may be an alternative treatment for AD with fewer side effects. A previous study showed that the phytoestrogen, α-zearanol, elevated the cell survival of Aβ (25–35)-induced PC-12 cells by attenuating oxidative stress and apoptotic cell death in a manner similar to 17β-estradiol [27]. In the present study, both S-equol and 17β-estradiol were also found to increase cell survival followed by Aβ (25–35) treatment. These results would predict that phytoestrogen, S-equol, possessed putative neuroprotective effects against Aβ (25–35)-induced cytotoxicity on SH-SH5Y cells analogous to those of 17β-estradiol [28,29]. In addition, the result of the inhibition of ER with antagonist ICI-182,780 prior to the Eq and E2 treatments suggested that ER may have a role in the neuroprotection of S-equol and 17β-estradiol against Aβ (25–35) cytotoxicity. The critical roles of ERs have been implicated in the cognitive function [14]. The loss of ERα expression has been noted to more likely contribute to AD-related memory impairment and amyloidogenesis [30,31]. Our observations showed the downregulation of ERα protein expression in SH-SH5Y cells exposed to Aβ (25–35) alone, emphasizing the importance of the ERα functional role in response to Aβ (25–35)-induced cytotoxicity. Under normal conditions, the ERα function can be enhanced by its coactivators, such as SRC-1, for efficient transcriptional regulation [32]. The decreased SRC-1 protein expression in the Aβ (25–35)-treated group was seen in the present study showing the Aβ (25–35)-induced disruption of the SRC-1 coactivator. These results support the notion that Aβ (25–35)-induced perturbation of ERα was further evident from the corresponding decrease in the expression of SRC-1. Furthermore, S-equol or 17β-estradiol pretreatments efficiently attenuated the effects of Aβ (25–35) in the current study, demonstrating that to provide a neuroprotective effect, equol binds with ERα and recruits SRC-1 to enhance its effect. On the other hand, the actions of both compounds were blocked by anti-estrogen ICI-182,780 in the present study, observing that ERα is required for the neuroprotective response of S-equol or 17β-estradiol to Aβ (25–35) cytotoxicity.

17β-estradiol binding to ERα is able to trigger transcriptional regulation of target genes, such as cyclin D1 [33]. In this regard, a recent study has reported that 17β-estradiol bound ERα has a role in controlling cell cycles [34]. In the present data, we presume that downregulated ERα expression in the presence of Aβ (25–35) might partially contribute to aberrant cell cycles. In normal conditions, neuron cells are postmitotic and stay in the G_0_ phase, as indicated by the downregulation of proteins related to the cell cycle [35]. For instance, cyclin D1, a protein marker of the G_0_/G_1_ phase, is expressed at the beginning of the G1 phase and continually accumulates in the nucleus during the G1 phase in the presence of the cell cycle reactivation [36]. When the cells progress into the S phase, cyclin D1 can secrete into the cytoplasm and its overexpression can reduce cell sizes and shorten the G1 phase resulting in the accelerated entry into the S phase [37]. Likewise, our results showed that Aβ (25–35) caused cells to leave the postmitotic phase and reenter the cell cycle in parallel with the increasing level of cyclin D1. This finding is in line with previous studies which found that Aβ (25–35) toxicity induces cell-cycle reentry [9,38]. However, only a tendency toward a decrease in cell number of the G1 phase in the Aβ-treated group was observed in this study. Such observation might be ascribed to a more rapid cell cycle progression in response to a higher level of cyclin D1 followed by Aβ treatment as mentioned above. Alternatively, it is plausible that there is a high degree of variability in the G1-phase progression due to the differences in nature between cells, which indicates that the cell itself may enter into G1 or exit from G1 at different time points from its neighboring cells [39]. In presenilin (PS)-1 familial AD brains, the presence of cyclin D1 accumulation was observed to be linked to cell-cycle activation and subsequently led to cell death [40]. Our results are in accordance with previous findings showing that when exposed to 25 μM Aβ (25–35), SH-SY5Y cells accumulated in the S phase, indicating that they did not progress beyond the S phase accompanied by apoptosis [9,41]. Taken together, we speculate that changes in ERα and cyclin D1 expressions concomitantly occurring with aberrant cell cycle reentry appear likely to underlie the cytotoxic mechanisms of Aβ (25–35). Thus, apoptotic neuronal death is presumably the consequence of Aβ (25–35)-induced cytotoxicity [42]. However, it is noteworthy that more recent evidence indicates neuronal cell death triggered by a cell cycle reentry event could be independent of an apoptotic mechanism in AD [43]. More in-depth investigation is warranted to resolve this discrepancy. Nevertheless, S-equol prevented Aβ (25–35)-induced changes in the cell-cycle behavior, ERα, and cyclin D1 expressions, indicative of the neuroprotective potential of S-equol. 

A common target for estrogen signaling and Aβ neurotoxicity is ERK 1/2 [9,10,44]. It was shown that ERK 1 and 2 are expressed in the pooled cerebrospinal fluid (CSF) of patients with AD, and elevated levels of ERK 1/2 in CSF are accompanied by increased levels of tau protein and the Aβ42 peptide [45]. Rapid activation of ERK 1/2 was reported in SH-SY5Y neuroblastoma cells exposed to Aβ (25–35) [9] and in mature hippocampal neurons [46]. Aberrant activation of ERK 1/2 was correlated with an elevated level of cyclin D1 that has been shown to be responsible for cell cycle reentry in neurons under Aβ-induced toxicity conditions, thereby potentiating the neuronal apoptosis responses [38]. The present data showed that Aβ (25–35) triggered ERK 1/2 activation, and pretreatments of S-equol and 17β-estradiol were able to prevent this response. In contrast, treatment with ICI-182,780 appeared to diminish the protective effects of S-equol and 17β-estradiol. These observations led us to propose that the neuroprotective mechanisms of the actions of S-equol and 17β-estradiol against Aβ (25–35) cytotoxicity might be mediated by the ERK1/2 pathways via ERα. Previous studies have shown that estrogen prevents cytotoxic effects of Aβ by activating MAPK which regulates ERK 1/2 expression and cyclin D1 to control cell cycle reentry [9,29]. Herein, we have shown that S-equol exhibited neuroprotective effects that mimicked the action of 17β-estradiol on Aβ (25–35)-treated SH-SY5Y cells through preventing cell cycle reentry downregulating cyclin D1 and ERα-mediated ERK 1/2 expressions, all of which might have involved suppression of Aβ (25–35)-induced cell cycle reentry by S-equol or 17β-estradiol pretreatments in the current study.

## 5. Conclusions

This study concludes that Aβ (25–35) caused diminished ERα levels, which mediated estrogen actions to disrupt normal cell cycle regulation and thus potentiates cell death. S-equol might act as a putative neuroprotective agent against Aβ (25–35) cytotoxicity, and its neuroprotective role might be, at least in part, attributed to its estrogenic potency. The observed putative neuroprotective effects of equol were associated with sustaining ERα levels and cell survival in our cell models. Furthermore, the molecular mechanism underlying this putative neuroprotection of S-equol is shown to involve the suppression of cell cycle reentry which might be synergized with ERα-involved activation of ERK 1/2 along with the prevented activation of cyclin D1.
